# Magnitude of undernutrition and its association with dietary diversity among older persons in Ethiopia: a systematic review and meta-analysis, 2023

**DOI:** 10.1017/jns.2023.84

**Published:** 2023-09-26

**Authors:** Getachew Sale Mezgebu, Legesse Petros, Etaferaw Alemayew, Gashaw Abebaw, Fentaw Wassie Feleke

**Affiliations:** 1 Department of Human Nutrition, School of Human Nutrition and Food Science Technology, College of Agriculture, Hawassa University, PO Box 05, Hawassa, Ethiopia; 2 Department of Public Health, School of Public Health, College of Medicine and Health Sciences, Woldia University, PO Box 400, Woldia, Ethiopia

**Keywords:** Dietary diversity, Malnutrition, Meta-analysis, Older persons, Systematic review, AOR, adjusted odd ratio, CDC, Centers for Disease Control and Prevention, CI, confidence interval, DDS, Dietary Diversity Score, FAO, Food and Agriculture Organization, IDA, iron deficiency anaemia, JBI, Joanna Briggs Institute, LIC, low-income country, MeSH, Medical Subject Headings, NGO, Non-Governmental Organization, PRISMA, Preferred Reporting Items for Systematic Review and Meta-Analysis, SNNP, Southern Nations, Nationalities, and People's Region, SSA, sub-Saharan Africa, WHO, World Health Organization

## Abstract

Undernutrition in elders remains under-detected, under-treated, and under-resourced and leads to further weight loss, increased infections, and delay in recovery from illness as well as increased hospital admissions and length of stay. The reports of the findings were fragmented and inconsistent in Ethiopia. Therefore, the main objective of this meta-analysis was to estimate the pooled prevalence of undernutrition and its association with dietary diversity among older persons in Ethiopia. Online databases (Medline, PubMed, Scopus, and Science Direct), Google, Google Scholar, and other grey literature were used to search articles until the date of publication. The Preferred Reporting Items for Systematic Reviews and Meta-Analyses guideline was followed. The random effect model was used to estimate the pooled prevalence; whereas subgroup analysis and meta-regression were performed to identify the probable source of heterogeneity using Stata version 14.0 software. Out of 522 studies accessed, 14 met our criteria and were included in the study. A total of 7218 older people (aged above 60 years old) were included in the study. The pooled proportion of undernutrition among older persons in Ethiopia was 20⋅6 % (95 % CI 17⋅3, 23⋅8). Elders who consumed low dietary diversity scores were strongly associated with undernutrition among older persons. Therefore, promoting appropriate intervention strategies for elders to improve dietary diversity practices and nutritional status is crucial.

## Introduction

Malnutrition is a condition where energy, protein, and other nutrients are insufficient, excessive, or imbalanced to sustain health, promote cell and tissue growth, and normal organ function, causing detrimental effects on body shape, function, and clinical outcomes.^(1)^

Elderly people over the age of 60 years are growing rapidly in developing countries. By 2030, the elderly population in the developing world is projected to grow 140 %.^(2)^ The elderly population in developing countries is growing more rapidly than in more developed nations and it is projected that in 2020, 70 % of those aged above 60 years will live in developing countries.^(3)^ Fifteen percent of the world population and 4⋅7 % of the Ethiopian population is categorised as an elderly population aged ≥60 years.^(4)^

Globally, the prevalence of malnutrition among the older population ranges from 23 to 46 %.^(5)^ Countries like Germany, Netherlands, and Austria have about 20⋅1, 18⋅3, and 22⋅5 %, respectively, of their healthy older population becoming either underweight or overweight/obese.^(6,7)^ In sub-Saharan Africa, the prevalence of malnutrition among older adults and elders ranges from 2⋅9 to 48 %.^(8–10)^ In Ethiopia, undernutrition among the elderly population is a common health problem and its magnitude range from 15⋅7 to 40⋅2 %.^(11–24)^

Nutritional deficiencies in older persons have serious negative consequences which can increase morbidity and mortality, hospitalisation, development of ulcers, and infections.^(12)^ Involuntary weight loss can result in a reduction in the ability to care for oneself, loss of mobility and independence, and a poorer quality of life.^(25)^

Undernutrition is an important global issue currently, as it affects all people despite the geography, socioeconomic status, sex and gender, overlapping households, communities, and countries. Reduction of income and physical in capability in older adults increases vulnerability to food insecurity, which in turn predisposes to undernutrition.^(26)^ Geographic and psychosocial concerns can also affect dietary behaviours and nutritional status.^(27)^

There is a lack of community-based programmes and activities that screen for undernutrition and address those risk factors among vulnerable populations.^(28)^ In Africa, the elderly population is not considered a priority for targeting nutrition interventions, so the efficacy of different types of nutrition interventions has not been described in this population. Hence, only a handful of nutritionists work with older people, and there are even fewer practitioners who perform high-quality research in their homes. Indeed, nothing else has been attempted, aside from the disaster relief and supplementary feeding programs.^(29)^

Ethiopia has been implementing policy, program and strategies such as Food and Nutrition program (FNP), Immunization and Health Sector Transformation Plan i.e. focusing to improve children, adolescents, and maternal health and nutrition.^(30)^ However, undernutrition among elderly population is under detected and neglected area because the entire problem related with aging considered as fate of aging. Correspondingly, undernutrition among elders is a public health research to advocate and speck up for policy endorsers.^(31)^ Therefore, this systematic review and meta-analysis study aimed to assess the pooled prevalence of undernutrition and its association with dietary diversity among elderly population in Ethiopia.

## Methods

### Study setting

This systematic review and meta-analysis were conducted in Ethiopian setting. Ethiopia is one of Africa's populous nations having 119,851,500 people with an area of 1,100,000 km^2^ making it the 27th largest country in the world. It is projected that 4⋅7 % of the Ethiopian population is categorised as an elderly population aged ≥60 years.^(4)^ The country has nearly 78⋅7 % of the rural population with diversified religion and cultures.^(32)^ Through the implementation of the National Nutrition Program and the IYCF, the nation is collaborating with foreign partners to eliminate maternal and child undernutrition^(33)^; however, there is no intervention for Ethiopia's senior population.

### Searching strategies

This systemic review and meta-analysis were designed to estimate the pooled prevalence of undernutrition and identify its potential predictors in Ethiopia. Initially, meta-analysis and systematic reviews, including registered protocols, were searched to avoid duplications. It was confirmed that there was no any review and meta-analysis was conducted related to undernutrition among elderly population in Ethiopia. Then, we attempted to provide answers to the questions in our review: (1) what is the prevalence of undernutrition among the older population in Ethiopia? (2) What are the factors influencing undernutrition in older persons in Ethiopia? Published research reports of undernutrition and its associated factors were searched. Moreover, the authors further expanded, via hand, the search to include other unpublished sources. The authors systematically reviewed and analysed published research articles to determine the pooled prevalence of the undernutrition and its predictors in Ethiopia. To identify published articles, major databases such as PUBMED/MEDLINE, Cochrane library, Google, and Google Scholar were used. In addition, reference lists and free web-based search was conducted to retrieve other relevant materials which assessed the nutritional status of the elderly and its associated factors. The key term used in PubMed search was ‘prevalence’ OR ‘magnitude’ AND ‘undernutrition’ OR ‘malnutrition’ OR ‘nutritional status’ OR ‘malnourished’ AND ‘associated factors’ AND ‘elderly population’ OR ‘elderly’ OR ‘old age’ OR ‘aged’ AND ‘Ethiopia’ AND ‘dietary intake’ OR ‘dietary diversity’ OR ‘dietary practice’. The searching was conducted up to the date of publication. The authors followed the Preferred Reporting Items for Systematic Reviews and Meta-Analyses (PRISMA) guideline during the systematic review.^(34)^

### Inclusion criteria

*Study scope:* Studies were included if conducted among the older persons 60 years and above in Ethiopia and evaluated the prevalence of undernutrition and associated factors of undernutrition included in this systematic review and meta-analysis.

*Study design:* A cross-sectional study design was included.

*Language:* All articles published in English language were included.

*Population:* All studies conducted in Ethiopia were considered.

*Publication year:* Published articles since 2014 were included.

### Exclusion criteria

Studies difficult to access full text, not English language and studies which did not report specific outcomes for undernutrition were excluded.

### Operationalisation of the outcomes of the study

The study contained two objectives, namely, the magnitude of undernutrition and the effect of low dietary diversity on undernutrition in Ethiopia. The prevalence of undernutrition was computed by MNA (mini-nutritional assessment) tool developed by Nestle Nutrition Institute^(35)^ and BMI. The MNA tool was validated in developing setting including Ethiopia.^(36)^

For the second objective, the odds ratio was computed by using the binary formula and it was estimated in the form of logs of the odds ratio. Dietary diversity score (DDS) was classified as low when an older person consumes <5 food items per day; High dietary diversity score: when an older adult consumes ≥5 food items per day.

### Data extraction

The database search results were collected and duplicate articles were removed manually using Endnote (version 20.4.1). The authors used two stages of screening. Primarily, we screened the titles and abstracts based on the inclusion criteria. Secondly, the authors identified potentially relevant articles using titles and abstracts for further re-screening of its full article document. The relevance of the articles was evaluated based on their topic, objectives, and methodology as listed in the abstract. The abstracts were also assessed for agreement with the inclusion criteria. When it was unclear whether an abstract was relevant, it was included for retrieval. Data were extracted by two authors (GSM and FWF) independently using a standardised data extraction spreadsheet. Data extraction sheet included study characteristics such as: (1) Authors’ name, region, study year, publication year, study design, study setting, sample size, response rate, studies’ quality score, and sampling; (2) prevalence of undernutrition; and (3) dietary diversity corresponding with experiencing status of the event (undernutrition) was extracted. Those categorical variables tabulated (a, b, c, and d) with undernutrition during abstraction. Any disagreement between the two authors due to inclusion and data collection was solved by the second, third, and fourth authors (LP, EA, and GA) through discussion.

### Quality assessment (appraisal) of studies

The database search results were combined and duplicate articles were removed manually using Endnote (version 20.4.1). Joanna Briggs Institute Meta-Analysis of Statistics Assessment and Review Instrument (JBI-MAStARI) adapted for both cross-sectional/cohort study design was used.^(37)^ Three independent reviewers critically evaluated each individual paper. Discrepancies between those reviewers were solved by discussion. If not, a third reviewer was involved to resolve inconsistencies in between the two independent reviewers. Primary studies that scored five and above from a total of 9 scores were selected for the final systematic review and meta-analysis. Studies, which measured undernutrition by using either MNA or BMI tool, were included in the final systematic review and meta-analysis.

### Data analysis

The extracted data were entered into an Excel sheet and imported to STATA version 14 for analysis. Heterogeneity among reported prevalence was assessed by using the inverse variance (*I*^2^) with Cochran Q statistic of 25, 50, and 75 % as low, moderate, and severe heterogeneity with a *P*-value of less than 0⋅05, respectively.^(38)^ Random effects meta-analysis model was used to estimate the pooled prevalence of undernutrition. The forest plot was also used to visualise the presence of heterogeneity graphically. Possible differences between the studies were explored by subgroup analyses and meta-regression. The finding was presented using the forest plot with respective odds ratio and 95 % confidence intervals. Evidence of publication bias was assessed using both Egger's and Begg's tests with a *P*-value of less than 0⋅05 as a cut-off point to declare the presence of publication bias.^(39,40)^ For the second outcomes, pooled odds ratios with 95 % CI for each factor were used to determine the association between undernutrition and dietary diversity practice.

## Results

### Selection of the studies

**Fig. 1.**
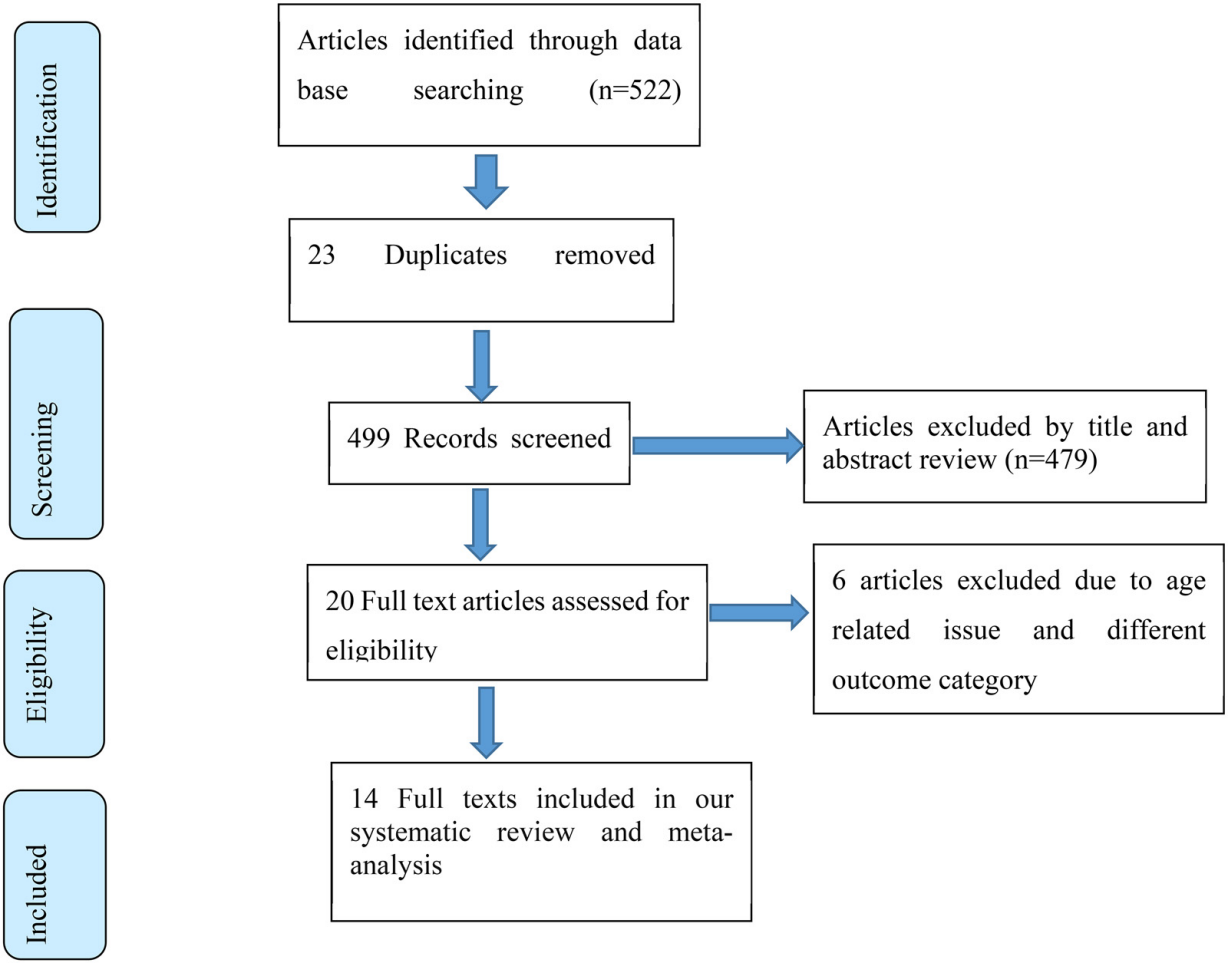
PRISMA flowchart diagram describing selection of studies for systematic review and meta-analysis on the prevalence of malnutrition and its association with dietary diversity among Ethiopian elderly population, a systematic review and meta-analysis, 2023.

Initially, a total of 522 studies were obtained through searching from different databases. Out of 522 studies, 23 studies were excluded because of duplication, and 479 studies were excluded after reading the title and abstract using inclusion and exclusion criteria. The remaining twenty full-text studies were assessed for eligibility and of which, six articles were excluded due to age-related issues and different outcome categories. Finally, fourteen studies were included in the systematic review and meta-analysis (Fig. 1).

### Characteristics of the studies

All 14 studies included in this systematic review and meta-analysis were done among 7218 elders in the six regions of Ethiopia. With regard to regional distribution, nearly half (42⋅86 %) of the studies were conducted in Amhara region.^(11,12,15,19,20,24)^ The prevalence of undernutrition ranged between 15⋅7 % in Harari^(18)^ to 40⋅2 % in Dire Dawa.^(23)^ With respect to the distribution of studies, three of the individual studies were obtained from Addis Ababa and Dire Dawa regions,^(23,31)^ two from Oromia,^(41,42)^ two from Harari region,^(18,22)^ and other two studies from the Southern Nations, Nationalities, and People's (SNNP) Region.^(13,16)^ However, there were no studies from Benishangule Gumize region, Gambela region, and Tigray region (Table 1).

**Table 1.** List of studies included to show the prevalence of undernutrition among Ethiopian elderly population 2013–2022, a systematic review and meta-analysis, Ethiopia, 2023

Authors	Study area	Study setting	Study year	Publication year	Measurement tool	Sample size	Study design	Sampling methods	Prevalence
Tessfamichael *et al.*^(11)^	Amhara	Community based	2013	2014	BMI	757	Cross-sectional	Multistage	21⋅9
Mezemir *et al.*^(19)^	Amhara	Community based	2020	2020	MNA	347	Cross-sectional	Simple random	21⋅1
Adhane *et al.*^(12)^	Amhara	Community based	2015	2019	MNA	423	Cross-sectional	Systematic random	22⋅7
Abate *et al.*^(17)^	Addis Ababa	Community based	2020	2020	MNA	682	Cross-sectional	Systematic random	26⋅6
Abdu *et al.*^(18)^	Harari	Community based	2019	2020	MNA	630	Cross-sectional	Multistage	15⋅7
Ferede *et al.*^(21)^	Oromia	Community based	2021	2022	MNA	616	cross-sectional	Multistage	17⋅5
Legesse *et al.*^(15)^	Amhara	Community based	2018	2019	BMI	921	cross-sectional	Census	17⋅6
Mulugeta *et al.*^(22)^	Harari	Community based	2020	2022	MNA	343	Cross-sectional	Simple random	16⋅6
Oumar *et al.*^(23)^	Dire Dawa	Facility based	2020	2022	MNA	164	Cross-sectional	Simple random	40⋅2
Wondiye *et al.*^(16)^	SNNP	Community based	2017	2019	BMI	578	Cross-sectional	Multistage	17⋅1
Yisak *et al.*^(24)^	Amhara	Community based	2020	2021	BMI	300	Cross-sectional	Systematic random	27⋅6
Hailemariam *et al.*^(13)^	SNNP	Community based	2012	2016	MNA	548	Cross-sectional	Systematic random	28⋅3
Abera *et al.*^(20)^	Amhara	Community based	2022	2022	MNA	289	Cross-sectional	Systematic random	34⋅8
Sheremu *et al.*^(42)^	Oromia	Community based	2021	2033	MNA	620	Cross-sectional	Simple random	17⋅4

### Pooled prevalence of undernutrition among elderly population in Ethiopia

The meta-analysis result in the forest plot showed that the *I*^2^ consistence test and Cochrane *Q* heterogeneity test statistics showed high heterogeneity (*I*^2^ = 89⋅5, *P*-value < 0⋅001), which leads us Dersimonian and Liard random effect model. As a result of a random effect model, the pooled proportion of undernutrition among elderly population in Ethiopia was 22⋅7 % (95 % CI 19⋅7, 25⋅6).

**Fig. 2.**
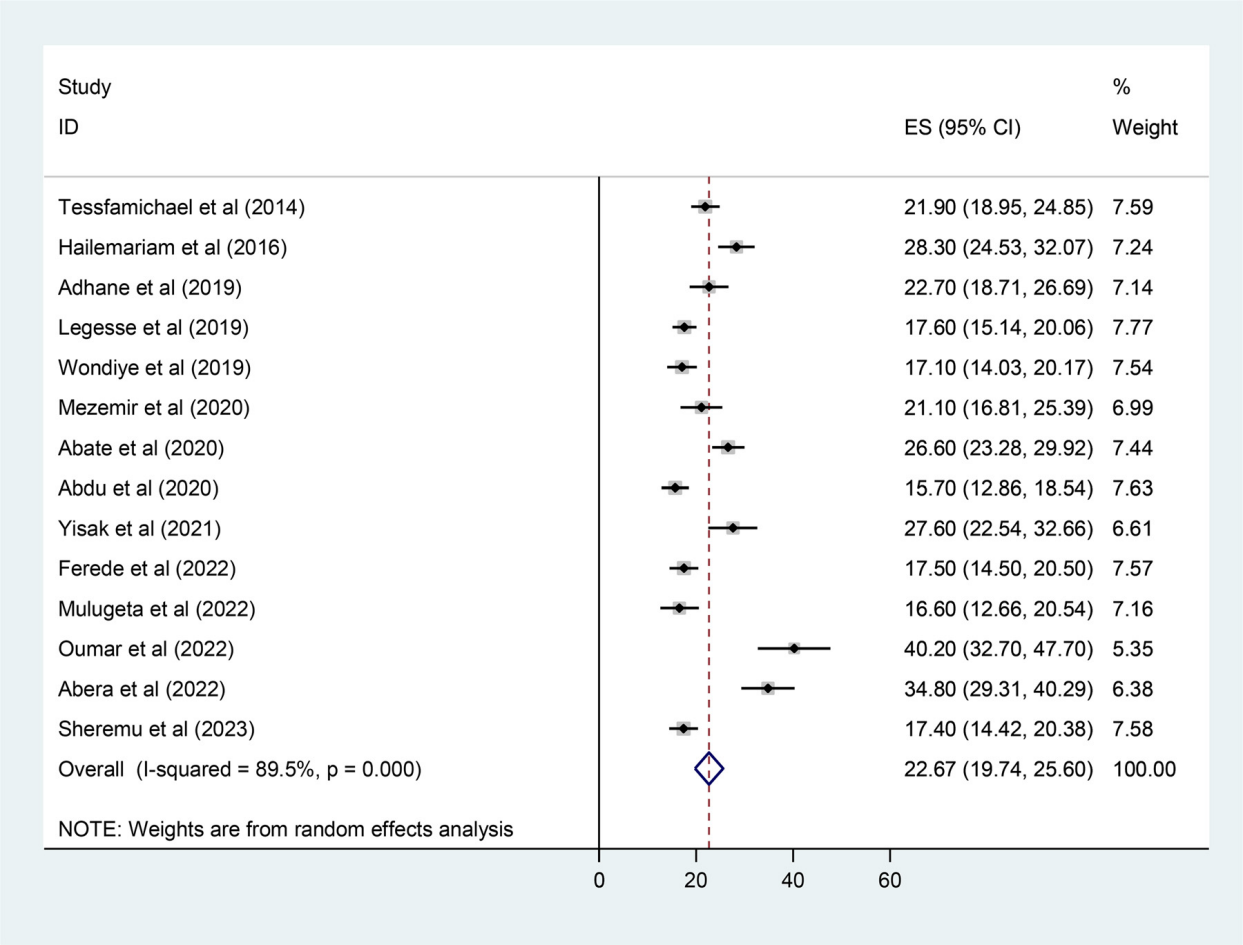
Forest plots showing the pooled prevalence of undernutrition among Ethiopian elderly population, a systematic review and meta-analysis, Ethiopia, 2023.

The significant magnitude of the heterogeneity also suggests the need to conduct subgroup analysis to identify the sources of heterogeneity (Fig. 2).

### Subgroup pooled prevalence analysis

Subgroup analysis was conducted to identify the possible source of heterogeneity and the distribution of undernutrition across studies based on regions, study years, sampling techniques, and nutritional status assessment tools. The subgroup analysis result directed that the source of heterogeneity was due to the study area, which implicates that Harari region has a lower prevalence of undernutrition was 16⋅0 % (95 % CI 13⋅7, 18⋅3) with a *P*-value of 0⋅716. However, the result directed that the source of heterogeneity was not due to the study years, sampling techniques nutritional status assessment tools, and study setting (*P* < 0⋅001) (Table 2).

**Table 2.** Subgroup analysis which indicates the pooled prevalence of undernutrition among Ethiopian elderly population, a systematic review and meta-analysis, Ethiopia, 2023

Subgroups		Number of studies	Prevalence (95 % CI)	Heterogeneity statistics	*P*-value	*I*^2^	Tau-squared
Survey year	Before 2020	5	21⋅37(17⋅60, 25⋅16)	28⋅82	<0⋅001	86⋅0 %	15⋅84
	Since 2020	9	23⋅61(19⋅18, 28⋅05)	95⋅73	<0⋅001	91⋅6 %	41⋅05
Region	Amhara	6	23⋅89(19⋅66, 28⋅14)	38⋅08	<0⋅001	86⋅9 %	23⋅72
	Oromia	2	17⋅5(15⋅33, 19⋅57)	0⋅00	0⋅963	0⋅0 %	0⋅00
	Harari	2	16⋅01(13⋅70, 18⋅31)	0⋅13	0⋅716	0⋅0 %	0⋅00
	SNNP	2	22⋅64(11⋅67, 33⋅62)	20⋅38	<0⋅001	95⋅1 %	59⋅64
	Others^a^	2	32⋅97(19⋅67, 46⋅27)	10⋅56	<0⋅001	90⋅5 %	83⋅72
Measurement tool	MNA	10	22⋅67(19⋅74, 25⋅60)	104⋅23	<0⋅001	91⋅4 %	38⋅02
	BMI	4	20⋅61(16⋅79, 24⋅43)	17⋅04	0⋅001	82⋅4 %	12⋅16
Sampling techniques	Systematic random	5	27⋅69(24⋅33, 31⋅05)	12⋅71	0⋅013	68⋅5 %	9⋅88
	Simple random	4	22⋅67(19⋅74, 25⋅60)	33⋅74	<0⋅001	69⋅0 %	5⋅09
	Other^b^	5	17⋅94(15⋅15, 19⋅89)	9⋅77	0⋅044	91⋅1 %	47⋅54
Sample size	<384	5	27⋅72(19⋅80, 35⋅61)	49⋅60	<0⋅001	91⋅9 %	73⋅90
	≥384	9	20⋅41(17⋅63, 23⋅20)	58⋅43	<0⋅001	86⋅3 %	15⋅55

a Dire Dawa and Addis Ababa.

b Census and multistage sampling.

The highest and lowest pooled prevalence of undernutrition among elderly population were observed in Harari region and others (Dire Dawa and Addis Ababa) region of Ethiopia were 16⋅0 % (95 % CI 13⋅7, 18⋅3) and 32⋅9 % (95 % CI 19⋅7, 46⋅3) with statistically significant heterogeneity indicating that the use of random effects models for estimating the pooled estimates is applicable, respectively (Table 2).

With respect to the study period, there was an increment of the pooled prevalence of undernutrition since 2020 from 21⋅4 % (95 % CI 17⋅6, 25⋅2) to 24⋅5 % (95 % CI 19⋅5, 29⋅6) with statistically significant heterogeneity indicating that the use of random effects models for estimating the pooled estimates is applicable.

The current study shows recorded the pooled prevalence of undernutrition among older persons using BMI and MNA tool measures to be 20⋅6 % (95 % CI 16⋅8, 24⋅4) and 22⋅7 % (95 % CI 19⋅7, 25⋅6), respectively.

**Fig. 3.**
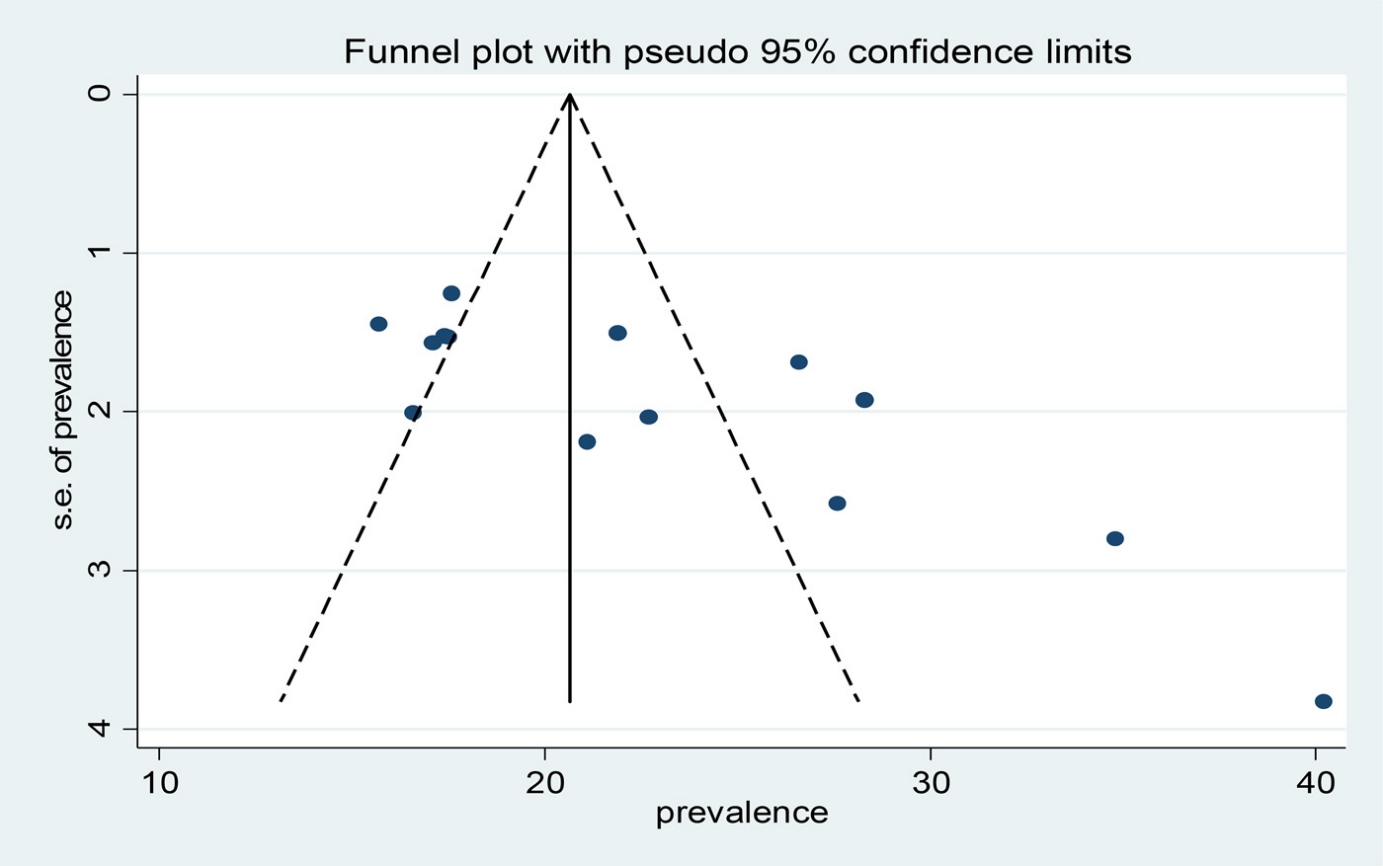
Funnel plot for the proportion of undernutrition Ethiopian elderly population, a systematic review and meta-analysis, 2023.

Publication bias as the source of heterogeneity was also checked using both Begg's and Egger's tests besides subgroup analysis. Based on Begg's and Egger's analyses, there are identified publication bias with a *P*-value of 0⋅014 and 0⋅001, respectively. In addition, the funnel plot seems asymmetry (Fig. 3).

**Fig. 4.**
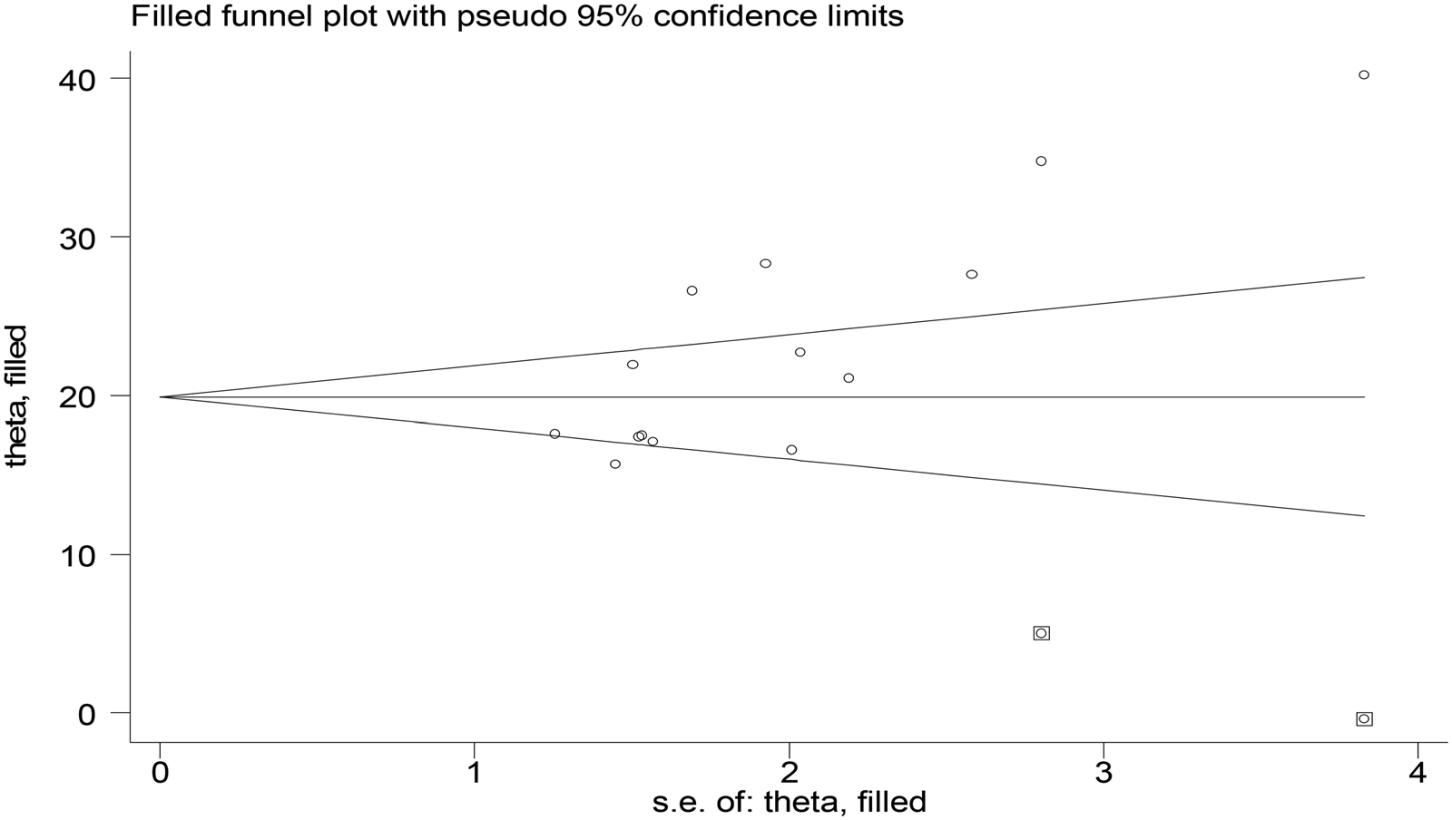
Trim and fill analysis for the proportion of undernutrition among Ethiopian elderly population, a systematic review and meta-analysis, 2023.

Furthermore, the trim and fill analysis was done to see the influence of the small study effect (Fig. 4). This method can indicate the positive studies that cause funnel plot asymmetry by conservatively imputing hypothetical negative unpublished studies. The adjusted summary pooled prevalence was based on the final result of the filled funnel plot (20⋅6 %, 95 % CI 17⋅3, 23⋅8, *P*-value < 0⋅001), which had varied substantially.

### Meta-regression

To determine the likely sources for the variation, we checked the potential factors associated with the prevalence variation, publication year, and sample size by using univariate meta-regression models but both of them were found to be statistically insignificant for the variation (Table 3).

**Table 3.** Meta-regression to identify source of heterogeneity for the prevalence of undernutrition among Ethiopian elderly population, a systematic review and meta-analysis, 2023

Variables	Coefficients	*P*-value
Sample size	−0⋅0194038	0⋅057
Publication year	0⋅131627	0⋅876

### Association of low dietary diversity with undernutrition

To examine the association between low dietary diversity score and undernutrition, only six studies with a total of 3266 Ethiopian elderly population were included. Four community-based studies from Amhara region^(11,15,20,24)^ and two community-based studies from Harari region^(22)^ and Metu, Oromia Region^(21)^ reported the dietary diversity as a factor of undernutrition. Out of the six studies, five studies reported that low dietary diversity were significantly and positively associated with undernutrition.

**Fig. 5.**
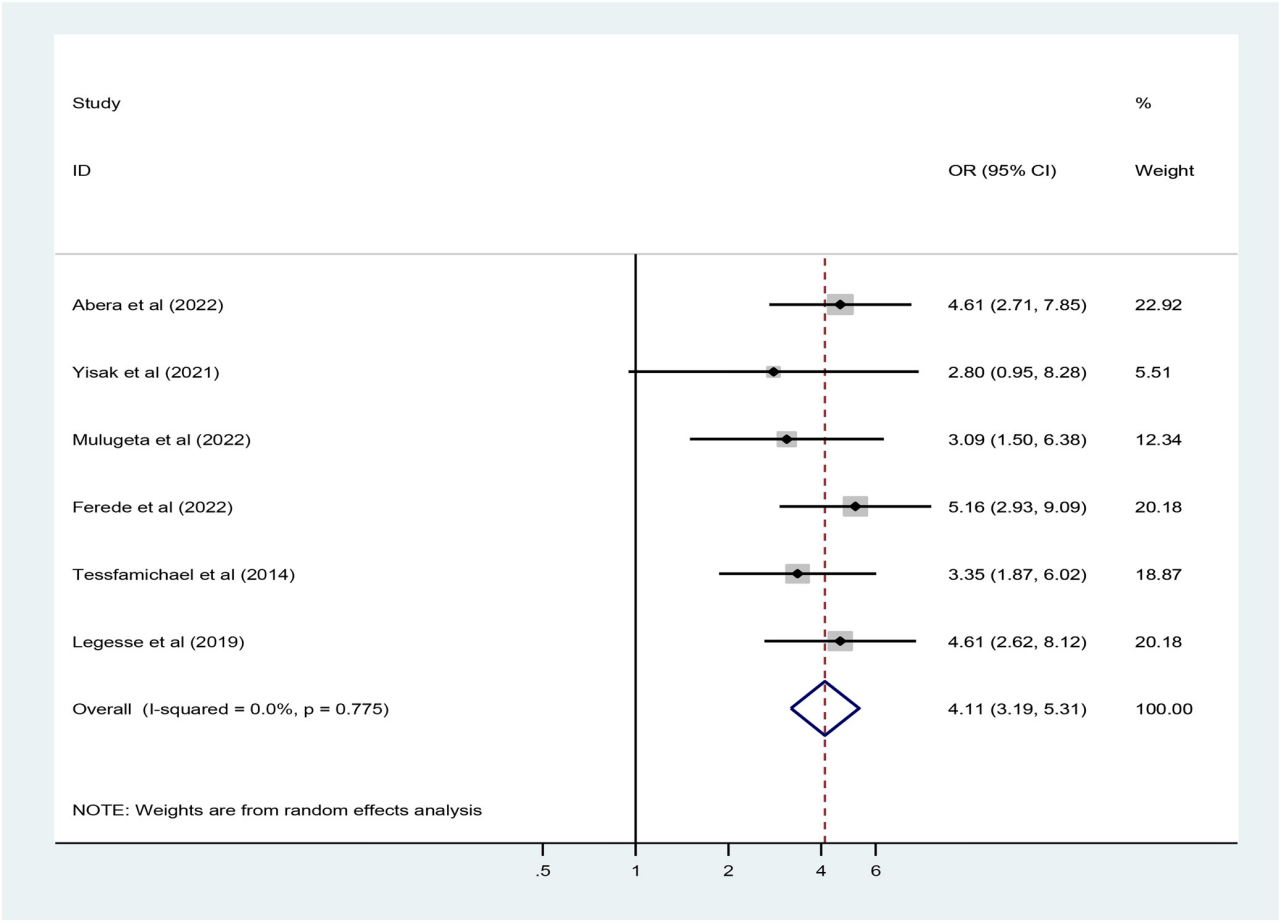
The pooled odds ratio of the association between low dietary diversity score and undernutrition among Ethiopian elderly population, a systematic review and meta-analysis, 2023.

**Fig. 6.**
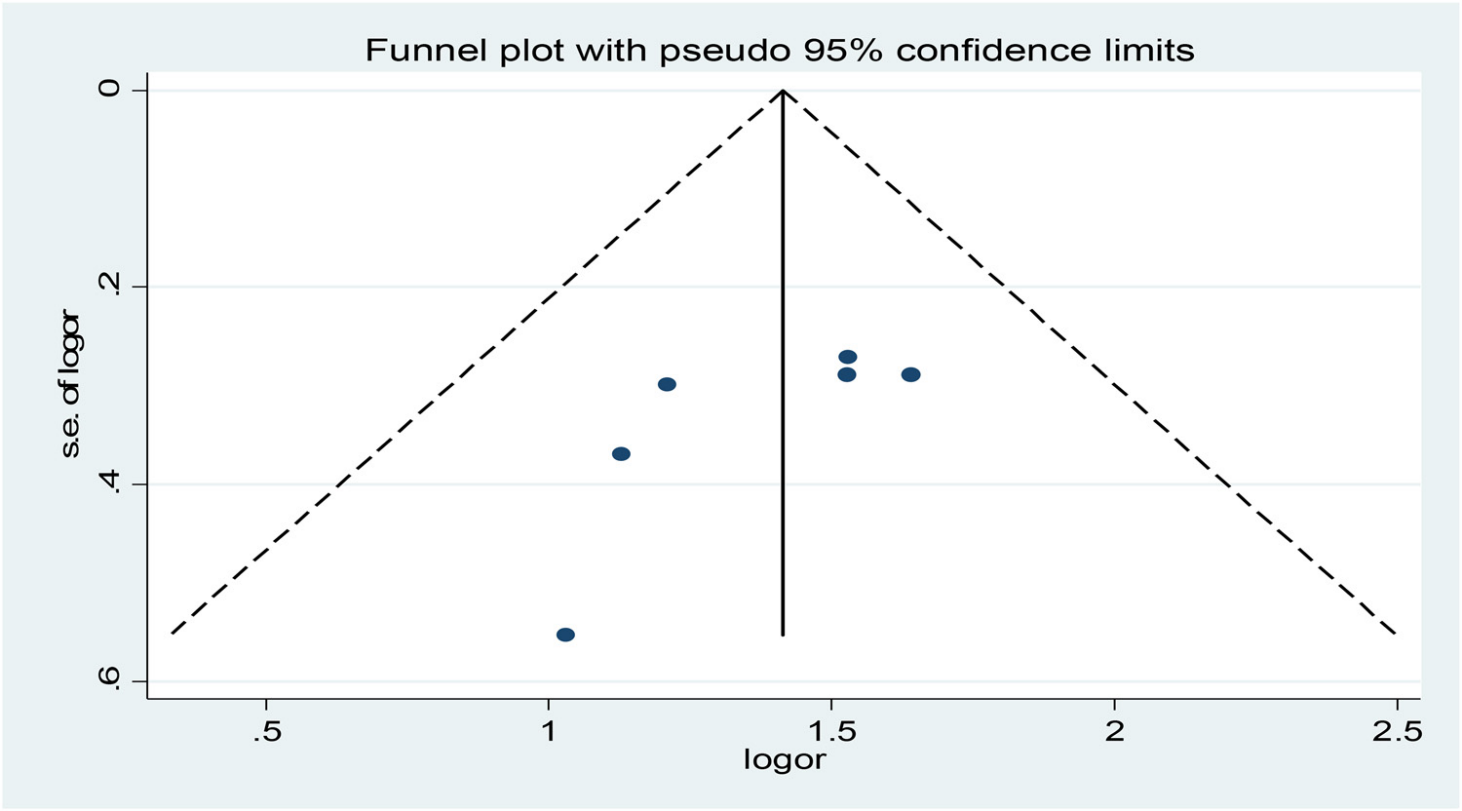
Funnel plot for odds ratio of the association between low dietary diversity score and undernutrition among Ethiopian elderly population, a systematic review and meta-analysis, 2023.

In the random effect model, weight was allocated based on the sample size and the effect size. The maximum (22⋅83 %) and minimum (18⋅22 %) weight were given for Abera *et al.*, and Tesfamichael *et al.*, respectively.^(11,20)^ The *I*^2^ consistence test and Cochrane *Q* heterogeneity test statistics showed high heterogeneity (*I*^2^ = 0 %, *P*-value = 0⋅775), which leads us Dersimonian and Liard random effect model. As an illustration, pooled odds of undernutrition among elderly population who consumed low dietary diversity score were increased by 4⋅1 (95 % CI 3⋅2, 5⋅3) folds as compared to those who consumed high dietary diversity scores (Fig. 5). Publication bias as the source of heterogeneity was checked using Egger's test. The funnel plot seems asymmetry but Egger's rank correlation publication bias test was insignificant (*P* = 0⋅126) (Fig. 6).

## Discussion

This systematic review and meta-analysis study aimed at determining the pooled prevalence of undernutrition and its association with low dietary diversity practice of older persons in Ethiopia.

The result showed that the pooled prevalence of undernutrition among elderly population in Ethiopia was 20⋅6 % (95 % CI 17⋅3, 23⋅8). The proportion was higher when compared to a study done in developing countries like Nigeria the prevalence was recorded to be 7⋅8 %,^(43)^ Ghana 18 %,^(44)^ Cameron, 19⋅7 %,^(45)^ Brazil, 18⋅8 %,^(46)^ Southwest China, 3⋅2 %,^(47)^ and Lebanon, 8 %.^(3)^ This increased prevalence of undernutrition in the study could be related to the study area's lower socioeconomic position, which could be associated with the lower food purchasing power of participants who varied their diet items.^(26,48)^ Furthermore, despite their increasing number and nutritional demands, little attention has been paid to the elderly, and undernutrition among the old population is an underdiagnosed and underserved area since the entire problem associated with aging is viewed as the fate of age.

In contrary, the result was lower when compared to a nutritional assessment conducted in the Central African Republic (CAR) among older persons found that 29⋅5 % were undernourished^(49)^ and Democratic Republic of Congo 28⋅4 %^(50)^ and the systematic review and meta-analysis study done in sub-Saharan Africa 28⋅4 %^(10)^ and a global prevalence of undernutrition among older persons report which ranges from 23 to 46 %.^(5)^ This could be attributed to the elderly population's poor screening habits, which are completely ignored due to their age in undeveloped nations such as Ethiopia.

The highest prevalence of undernutrition among elderly population was observed in other (Dire Dawa and Addis Ababa) region of Ethiopia followed by Amhara region while the lowest magnitude was observed in Harari region. This may be due to geographical location, production, availability, and cultural preference across regions in Ethiopia. Furthermore, this could imply that dietary practice attribution to nutrition status has a considerable impact on dietary variety in different agro-ecological zones, as factors vary by area.^(51)^

The current study recorded the pooled prevalence of undernutrition among older persons using BMI and MNA tool measures to be 20⋅6 and 22⋅7 %. As the MNA nutritional assessment tool was validated in different study areas of Ethiopia^(13,14,23,52)^ and pointed out a reliable and valid nutrition assessment method for identifying undernutrition, the result implicates that BMI measurement might underestimate the magnitude of undernutrition.

DDS is an important measure of individual's diet quantity and quality, and thus poor DDS may result in poor nutritional outcomes.^(53)^ In addition, DDS is associated with the economic ability of an individual to afford and access for varied types of food groups and better nutritional status.^(54)^ Therefore, diversity in the diet is important to meet the requirements for energy and other essential nutrients especially for those who are in the risk of nutrition deficiencies.^(55)^

The current review explored that the pooled odds of undernutrition among elderly population who consumed low dietary diversity score were increased by four folds as compared to those who consumed high dietary diversity scores. This finding was supported by findings conducted in sub-Saharan Africa studies,^(10,53)^ Zambia^(56)^ and Kenya.^(57)^ This might be due to the fact that a change in dietary diversity could cause a change in nutritional status.^(56)^ Furthermore, low calories and less protein content of less diversified diet result under nutrition, this implicates that there is a need to intervene on the improvement of dietary diversity among this age group.

Lack of dietary diversity is particularly a considerable problem among poor populations of the developing world as their diets are predominantly based on starchy staples.^(56,58)^ The dietary diversity practice might vary because of the reference difference to calculate DDS, the number of food groups included in the score, production, and lack of accessibility to diversified diet like animal source foods, fruits, and vegetables through well-established markets, seasonal variability, variations in geographical location, socioeconomic, and cultural preference differences across the countries.

The result of this study implied that one of the fourth elderly population of Ethiopia is malnourished. As a result, there is a need to strengthen dietary diversity practice in our country. The findings of this study have a significant contribution to addressing the nutritional problems of elderly population.

### Strengths and limitations

This review and meta-analysis study is the first of its kind in Ethiopia and searching key terms was broader which increased the number of included studies as a result, generalisability is possible with great confidence. Nevertheless, this study is not free of limitations. Accordingly, the study shared all the drawbacks of the random effect model. Because of the lack of further subgroup definitions in the primary studies, the heterogeneity among studies could not be resolved. Moreover, it would have been very good if the study considered other factors which influence undernutrition.

## Conclusion

Undernutrition among elderly population in Ethiopia was found to be high. The highest magnitude was observed in Dire Dawa and Addis Ababa. Low dietary diversity was found to be statistically correlated with undernutrition among old ages. Therefore, advocating for the improvement of dietary diversity practice among Ethiopian elderly population is strongly recommended. More importantly, there should be a nutritional policy that will improve older persons’ dietary habits and financial support systems should be established and made available to support older persons. Special emphasis should be also given to some regions where the problem is highly prevalent. Moreover, caregivers of older persons need nutrition education to increase awareness of proper nutritional care.
